# Comprehensive ensemble in QSAR prediction for drug discovery

**DOI:** 10.1186/s12859-019-3135-4

**Published:** 2019-10-26

**Authors:** Sunyoung Kwon, Ho Bae, Jeonghee Jo, Sungroh Yoon

**Affiliations:** 1 Department of Electrical and Computer Engineering, Seoul National University, Seoul, 08826 South Korea; 2 Interdisciplinary Program in Bioinformatics, Seoul National University, Seoul, 08826 South Korea; 3 Clova AI Research, NAVER Corp., Seongnam, 13561 South Korea; 4 Biological Sciences, Seoul National University, Seoul, 08826 South Korea; 5 ASRI and INMC, Seoul National University, Seoul, 08826 South Korea; 6 Institute of Engineering Research, Seoul National University, Seoul, 08826 South Korea

**Keywords:** Ensemble-learning, Meta-learning, Drug-prediction

## Abstract

**Background:**

Quantitative structure-activity relationship (QSAR) is a computational modeling method for revealing relationships between structural properties of chemical compounds and biological activities. QSAR modeling is essential for drug discovery, but it has many constraints. Ensemble-based machine learning approaches have been used to overcome constraints and obtain reliable predictions. Ensemble learning builds a set of diversified models and combines them. However, the most prevalent approach random forest and other ensemble approaches in QSAR prediction limit their model diversity to a single subject.

**Results:**

The proposed ensemble method consistently outperformed thirteen individual models on 19 bioassay datasets and demonstrated superiority over other ensemble approaches that are limited to a single subject. The comprehensive ensemble method is publicly available at http://data.snu.ac.kr/QSAR/.

**Conclusions:**

We propose a comprehensive ensemble method that builds multi-subject diversified models and combines them through second-level meta-learning. In addition, we propose an end-to-end neural network-based individual classifier that can automatically extract sequential features from a simplified molecular-input line-entry system (SMILES). The proposed individual models did not show impressive results as a single model, but it was considered the most important predictor when combined, according to the interpretation of the meta-learning.

## Background

Quantitative structure-activity relationship (QSAR) is a computational or mathematical modeling method to reveal relationships between biological activities and the structural properties of chemical compounds. The underlying principle is that variations in structural properties cause different biological activities [1]. Structural properties refer to physico-chemical properties, and biological activities correspond to pharmacokinetic properties such as absorption, distribution, metabolism, excretion, and toxicity.

QSAR modeling helps prioritize a large number of chemicals in terms of their desired biological activities as an in silico methodology and, as a result, significantly reduces the number of candidate chemicals to be tested with in vivo experiments. QSAR modeling has served as an inevitable process in the pharmaceutical industry, but many constraints are involved [2, 3]. QSAR data may involve a very large number of chemicals (more than hundreds of thousands); each chemical can be represented by a variety of descriptors; commonly used fingerprints are very sparse (most of the values are zero), and some features are highly correlated; it is assumed that the dataset contains some errors because relationships are assessed through in situ experiments.

Due to these constraints, it has become difficult for QSAR-based model prediction to achieve a reliable prediction score. Consequently, machine learning approaches have been applied to QSAR prediction. Linear regression models [4] and Bayesian neural networks [5–7] have been used for QSAR prediction. Random forest (RF) [8, 9] is most commonly used algorithm with a high level of predictability, simplicity, and robustness. RF is a kind of ensemble method based on multiple decision trees that can prevent the overfitting from a single decision tree. RF is considered to be the gold standard in this field [2]; thus, newly proposed QSAR prediction methods ofen have their performance compared to RF.

The Merck Kaggle competition in 2012 turned people’s attentions to neural networks. The winning team used multi-task neural networks (MTNNs) [10]. The fundamental learning structure is based on plain feed-forward neural networks; it avoids overfitting by learning multiple bioassays simultaneously. The team obtained results that consistently outperformed RF. Despite achieving high performance using a multi-task neural network, the team ultimately used an ensemble that combined different methods.

Both RF and the aforementioned technique from the Kaggle competition used ensemble learning, a technique which builds a set of learning models and combines multiple models to produce final predictions. Theoretically and empirically, it has been shown that the predictive power of ensemble learning surpasses that of a single individual learner if the individual algorithms are accurate and diverse [11–14]. Ensemble learning manages the strengths and weaknesses of individual learners, similar to how people consider diverse opinions when faced with critical issues.

Ensemble methods, including neural network ensemble based on bootstrap sampling in QSAR (*data sampling ensemble*) [15]; ensemble against different learning methods for drug-drug interaction [16], Bayesian ensemble model with different QSAR tools (*method ensemble*) [7], ensemble learning based qualitative and quantitative SAR models [17], Hybrid QSAR prediction model with various learning methods [18], ensembles with different boosting methods [19], Hybridizing feature selection and feature learning in QSAR modeling [20], and ensemble against diverse chemicals for carcinogenicity prediction (*representation ensembles*) [21] have been extensively used in drug (chemical) research. However, these ensemble approaches limit model diversity to a single subject, such as data sampling, method, and input representation (drug-specific).

To overcome this limitation, we propose a multi-subject comprehensive ensemble with a new type of individual classifier based on 1D-CNNs and RNNs. The detailed key characteristics and contributions of our proposed methods are as follows:

- Instead of limiting ensemble diversity to a single subject, we combine multi-subject individual models comprehensively. This ensemble is used for combinations of bagging, methods, and chemical compound input representations.
- We propose a new type of individual QSAR classifier that is an end-to-end neural network model based on one-dimensional convolutional neural networks (1D-CNNs) and recurrent neural networks (RNNs). It automatically extracts sequential features from a simplified molecular-input line-entry system (SMILES).
- We combine a set of models using second-level combined learning (meta-learning) and provide an interpretation regarding the importance of individual models through their learned weights.

To validate our proposed method, we tested 19 bioassays specified in [10]. In our experiments, we confirmed the superiority of our proposed method by comparing individual models, limited ensemble approaches, and other combining techniques. Further, we identified the importance of the proposed end-to-end individual classifier through an interpretation of second-level meta-learning.

## Results

### Experimental setup

#### Dataset

A bioassay is a biochemical test to determine or estimate the potency of a chemical compound on targets and has been used for a variety of purposes, including drug development, and environmental impact analysis. In our experiment, we used 19 bioassays downloaded from the PubChem open chemistry database [22], which are listed in Table 1. All bioassays are those specified in [10]. The purpose of the paper was to address multi-task effects; thus, a number of experimental assays are closely related, such as the 1851, 46321*, 48891*, and 6517** series.

**Table 1:** Details of the bioassay datasets used in the experiments

Assay ID	Description of BioAssay	# Active	# Inactive
1851_1a2	Cytochrome P450 Panel Assay, cyp1a2	5,902	6,974
1851_2c19	Cytochrome P450 Panel Assay, cyp2c19	5,840	7,135
1851_2c9	Cytochrome P450 Panel Assay, cyp2c9	4,065	8,361
1851_2d6	Cytochrome P450 Panel Assay, cyp2d6	2,601	10,826
1851_3a4	Cytochrome P450 Panel Assay, cyp3a4	5,175	7,446
1915	Streptokinase Expression Inhibition	2,219	1,017
2358	Inhibitors of Protein Phosphatase 1 (PP1)	1,006	934
463213	Inhibitors of tim10-1 yeast	4,138	3,234
463215	Inhibitors of tim10 yeast	2,941	1,695
488912	Inhibitors of Sentrin-specific protease 8	2,491	3,705
488915	Inhibitors of Sentrin-specific protease 6	3,568	2,628
488917	Inhibitors of Sentrin-specific protease 7	4,283	1,913
488918	Inhibitors of Sentrin-specific proteases	3,691	2,505
492992	Inhibitors of KCNK9 ^∗^	2,094	2,820
504607	Inhibitors of Mdm2/MdmX interaction	4,825	1,406
624504	Inhibitors of the mtPTP ^*†*^	3,944	1,090
651739	Inhibition of T.cruzi proliferation	4,043	1,322
651744	NIH/3T3 (mouse embryonic fibroblast) toxicity	3,099	2,303
652065	Molecules that bind r(CAG) RNA repeats	2,965	1,286

The 19 bioassays are those specified in [10]

^∗^Two-pore domain potassium channel

^*†*^Mitochondrial permeability transition pore

From each bioassay, we extracted a PubChem chemical ID and activity outcome (active or inactive). We only used duplicate chemicals once, and we excluded inconsistent chemicals that had both active and inactive outcomes. A class imbalance ratio between active and inactive ranged from 1:1.1 to 1:4.2 depending on the dataset; most bioassays are imbalanced, with an average ratio of 1:2.

#### Representation of chemical compounds

In our experiment, we used three types of molecular fingerprints PubChem [22], ECFP [23], MACCS [24], and string type SMILES [25]. Because SMILES is a sequential string type descriptor, it is not a proper form for conventional learning methods. We used an end-to-end 1D-CNN and RNN which are capable of handling a sequential forms. On the other hand, a binary vector type fingerprint consists of 1’s and 0’s in a form of non-sequential form. Thus, conventional machine learning approaches such as plain feed-forward neural network are used.

The SMILES and PubChem fingerprint were retrieved from the preprocessed chemical IDs using PubChemPy [26], and ECFP and MACCS fingerprints were retrieved from SMILES using RDKit [27].

#### Experimental configuration and environment

We followed the same experimental settings and performance measures as described for the multi-task neural network [10]. We randomly divided the dataset into two parts: 75% of the dataset was used as a training set, and the other 25% was used as a testing set. The training dataset was also randomly partitioned into five portions: one for validation, and the remaining four for training (5-fold cross-validation). The prediction probabilities from the 5-fold validations were concatenated as *P*, and were then used as inputs for the second-level learning.

We ran our experiments on Ubuntu 14.04 (3.5GHz Intel i7-5930K CPU and GTX Titan X Maxwell(12GB) GPU). We used the Keras library package (version 2.0.6) for neural network implementation, the Scikit-learn library package (version 0.18) for conventional machine learning methods, and PubChemPy (version 1.0.3) and RDKit (version 1.0.3) for input representation preparation of the chemical compounds.

### Performance comparison with other approaches

#### Performance comparison with individual models

We compared our comprehensive ensemble method with 13 individual models: the 12 models from the combination of three types of fingerprints (PubChem, ECFP, and MACCS) and four types of learning methods (RF, SVM, GBM, and NN), and a SMILES-NN combination.

As shown in Table 2, the comprehensive ensemble showed the best performance across all datasets, followed by ECFP-RF and PubChem-RF. We can see that the top-3 AUCs (represented in bold) are dispersed across the chemical compound representations and learning methods, except for PubChem-SVM, ECFP-GBM, and MACCS-SVM. The individual SMILES-NN models were within the top-3 ranks of the three datasets. In terms of learning methodology, RF showed the highest number of top-3 AUC values followed by NN, GBM, and SVM. In terms of chemical compound representation, ECFP showed the highest number of top-3 AUC values followed by PubChem, SMILES (compared proportionally), and MACCS. In terms of the averaged AUC, the comprehensive ensemble showed the best performance (0.814), followed by ECFP-RF (0.798) and PubChem-RF (0.794). The MACCS-SVM combination showed the lowest AUC value (0.736). Aside from the best (proposed ensemble) and the worst (MACCS-SVM) methods, all average AUC values were less than 0.80. Predictability depends on the combination of learning method and input representation. Although SVM showed better performance than GBM in ECFP, GBM showed better performance than SVM in MACCS.

**Table 2:** Performance comparison between the proposed comprehensive ensemble and the individual models on 19 bioassay datasets

BioAssay	PubChem fingerprint				ECFP fingerprint				MACCS fingerprint				SMILES	comprehensive
	RF	SVM	GBM	NN	RF	SVM	GBM	NN	RF	SVM	GBM	NN	NN	ensemble
1851_1a2	**0.921**	0.896	0.900	**0.921**	0.919	0.906	0.882	0.920	0.912	0.879	0.894	0.912	**0.922**	**0.934**
1851_2c19	0.871	0.852	0.848	0.872	0.882	0.871	0.854	**0.880**	0.874	0.842	0.850	**0.885**	0.875	**0.900**
1851_2c9	0.871	0.857	0.851	0.873	**0.880**	0.866	0.843	**0.880**	0.858	0.828	0.840	0.870	0.877	**0.898**
1851_2d6	0.858	0.847	0.832	**0.869**	**0.867**	0.850	0.833	0.856	0.854	0.816	0.830	0.852	0.846	**0.884**
1851_3a4	0.877	0.868	0.865	0.887	**0.891**	0.887	0.855	**0.895**	0.867	0.832	0.851	0.875	**0.891**	**0.914**
1915	0.754	0.692	0.709	0.722	0.731	0.700	0.700	0.712	**0.758**	0.716	0.736	**0.741**	0.701	**0.755**
2358	**0.787**	0.705	0.736	0.770	0.780	0.767	0.722	0.761	0.774	0.731	0.763	**0.775**	0.697	**0.803**
463213	0.673	0.639	0.652	0.651	**0.685**	0.652	0.644	0.661	**0.668**	0.642	0.655	0.651	0.636	**0.689**
463215	0.620	0.576	0.592	0.604	0.617	0.585	0.598	0.595	**0.629**	0.600	**0.630**	0.625	0.587	**0.627**
488912	0.679	0.643	0.634	0.668	**0.693**	0.654	0.668	0.675	0.667	0.634	0.650	**0.673**	0.644	**0.698**
488915	0.718	0.686	0.679	0.713	**0.731**	0.693	0.680	**0.708**	0.692	0.659	0.680	0.693	0.679	**0.735**
488917	**0.808**	0.777	0.759	0.805	**0.814**	0.788	0.760	0.799	0.788	0.726	0.752	0.786	0.780	**0.834**
488918	0.762	0.745	0.735	**0.778**	**0.778**	0.766	0.729	0.767	0.737	0.690	0.708	0.742	0.746	**0.799**
492992	**0.829**	0.784	0.783	0.800	**0.849**	0.807	0.802	0.822	0.825	0.726	0.759	0.790	0.802	**0.845**
504607	**0.694**	0.678	**0.692**	0.686	0.690	0.668	0.673	0.656	0.676	0.640	0.662	0.655	0.649	**0.721**
624504	**0.884**	0.850	0.857	0.867	**0.884**	0.858	0.858	0.861	0.872	0.832	0.862	0.876	0.868	**0.897**
651739	0.791	0.770	0.773	0.781	**0.802**	0.782	0.771	0.788	0.779	0.729	0.759	0.754	**0.792**	**0.804**
651744	0.884	0.862	0.872	0.885	**0.889**	0.883	0.875	0.896	0.869	0.829	0.843	0.853	**0.899**	**0.901**
652065	**0.800**	0.752	0.782	0.780	**0.785**	0.775	0.758	0.774	0.776	0.736	0.759	0.772	0.763	**0.826**
average	**0.794**	0.762	0.766	0.786	**0.798**	0.777	0.763	0.784	0.783	0.741	0.762	0.778	0.771	**0.814**

Each value shows the averaged AUC from twenty repeated experiments on the test set (bold: top 3 AUC on each dataset), and the last row shows the averaged AUC calculated from 19 AUC results

Statistical analysis with paired *t*-tests was performed to evaluate differences between the means of paired outcomes. The AUC scores of the comprehensive ensembles were compared with the top-scored AUC from the individual classifier in each dataset from the five fold cross-validation. Assuming that two output scores *y*_1_ and *y*_2_ follow normal distributions, the difference between these two scores should also follow a normal distribution. The null hypothesis of no difference between the means of two output scores, calculated as *d*=*y*_1_−*y*_2_, indicates that the distribution of this difference has mean 0 and variance $\sigma ^{2}_{d}$. The comprehensive ensemble achieved an AUC score exceeding the top-scored AUC from an individual classifier in 16 out of 19 PubChem bioassays as shown in Table 3. Let $\bar {d}, s_{d}$, *n* denote the mean difference, the standard deviation of the differences, and the number of samples, respectively. The results are significant at a p-value of 8.2×10^−7^, where the t value is calculated by $t_{d} = \frac {\bar {d}} {\frac {s_{d}}{\sqrt {n}}} \sim t_{n-1}.$

**Table 3:** The AUC scores of the ensemble classifier and the best single classifier for 19 PubChem assays

Assay ID	The Best Single Classifier (AUC)	The Ensemble Classifier (AUC)
1851_1a2	0.922	0.934
1851_2c19	0.885	0.900
1851_2c9	0.88	0.898
1851_2d6	0.867	0.884
1851_3a4	0.895	0.914
1915	0.758	0.755
2358	0.787	0.803
463213	0.685	0.689
463215	0.630	0.627
488912	0.693	0.698
488915	0.731	0.735
488917	0.814	0.834
488918	0.778	0.799
492992	0.849	0.845
504607	0.694	0.721
624504	0.884	0.897
651739	0.802	0.804
651744	0.899	0.901
652065	0.800	0.826

#### Performance comparison with other ensemble approaches

In addition to a comparison with individual models, we compared the proposed ensemble method with other ensemble approaches based on the ensemble subject and combining technique, as shown in Table 4.

**Table 4:** Performance comparison with other ensemble approaches

BioAssay	limited ensemble								comprehensive ensemble	
	method ensemble			representation ensemble						
	PubChem	ECFP	MACCS	RF	SVM	GBM	NN	NN (+SMILES) ^∗^	average	meta-learning
1851_1a2	0.921	0.922	0.910	0.931	0.920	0.907	**0.937**	**0.941**	0.934	**0.943**
1851_2c19	0.875	0.889	0.879	0.893	0.887	0.869	**0.902**	**0.905**	0.900	**0.908**
1851_2c9	0.878	0.885	0.866	0.888	0.882	0.865	**0.899**	**0.905**	0.898	**0.908**
1851_2d6	0.870	0.869	0.853	0.880	0.869	0.852	**0.884**	**0.886**	**0.884**	**0.892**
1851_3a4	0.890	0.902	0.874	0.898	0.901	0.881	0.913	**0.919**	**0.914**	**0.920**
1915	0.729	0.721	0.750	**0.766**	0.728	0.739	0.747	0.750	**0.755**	**0.764**
2358	0.758	0.781	0.780	**0.805**	0.780	0.772	**0.805**	0.803	0.803	**0.807**
463213	0.669	0.672	0.669	**0.689**	0.671	0.666	0.682	0.684	**0.689**	**0.694**
463215	0.604	0.603	**0.639**	**0.636**	0.604	0.623	0.623	0.624	0.627	**0.634**
488912	0.674	0.682	0.676	**0.698**	0.668	0.667	0.695	**0.698**	**0.698**	**0.700**
488915	0.720	0.719	0.699	0.731	0.711	0.700	0.732	**0.737**	**0.735**	**0.739**
488917	0.811	0.815	0.785	0.824	0.808	0.782	0.832	**0.838**	**0.834**	**0.841**
488918	0.777	0.783	0.743	0.780	0.782	0.752	0.793	**0.799**	**0.799**	**0.801**
492992	0.820	0.829	0.795	**0.854**	0.818	0.812	0.836	0.845	**0.845**	**0.862**
504607	**0.710**	0.687	0.682	0.708	0.701	0.703	0.698	0.706	**0.721**	**0.726**
624504	0.879	0.875	0.867	0.896	0.880	0.878	0.892	**0.900**	**0.897**	**0.904**
651739	0.795	**0.806**	0.774	0.800	0.776	0.783	0.803	**0.807**	0.804	**0.809**
651744	0.892	**0.902**	0.868	0.890	0.882	0.879	0.899	**0.905**	0.901	**0.909**
652065	0.795	0.791	0.784	0.807	0.804	0.803	0.813	**0.822**	**0.826**	**0.832**
average	0.793	0.796	0.784	0.809	0.793	0.786	0.810	**0.814**	**0.814**	**0.821**

All AUC values except those in the last two columns are based on limited subject ensembles, while the AUC values in the last two columns are from the comprehensive ensemble. The first three columns are method ensembles that consider various methods by fixing them to a target molecular fingerprint. The next five columns are representation ensembles that consider various chemical compound representations by fixing them to a learning method. Except for the final meta-learning approach, combining is based on uniform averaging. Each value is the averaged AUC from five repeated experiments (bold: top 3)

^∗^NN(+SMILES) is a representation ensemble that combines a set of models trained on a diversified input representation of fingerprints (PubChem, ECFP, MACCS) and SMILES-based on NN

The first three columns showe the method ensemble, which combines predictions from RF, SVM, GBM, and NN by fixing them to a particular chemical representation. The ensembles based on PubChem, ECFP, and MACCS showed AUC values of 0.793, 0.796, and 0.784, which are 0.016, 0.015, and 0.018 higher than the average AUC value for the four individual methods based on those representations, respectively. The next five columns show the representation ensembles, which combine the PubChem, ECFP, and MACCS molecular representations by fixing them to a particular learning method. As with the method ensembles, the representation ensembles outperformed the average results from the individual representation models based on their learning methods. In particular, the NN-based individual models showed lower AUCs values than the RF-based models, but the NN-based combined representation ensemble showed a higher AUC value than the RF-based ensemble.

**Fig. 1:**
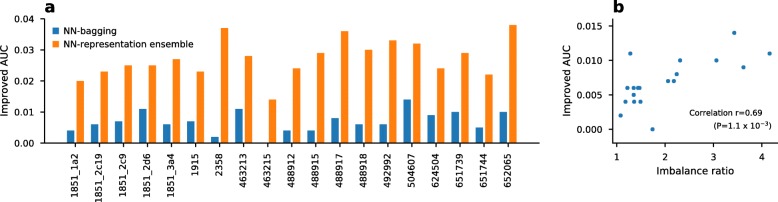
Ensemble effects on class-imbalanced datasets. **a** Improved average AUC value produced by neural network bagging (NN-bagging) and neural network-based representation ensemble (NN-representation ensemble) over three fingerprints. **b** Pearson’s correlation (r=0.69, p-value=1.1x 10^−3^) between the improved AUC values from NN-bagging and the class imbalance ratio. The class imbalance ratio was calculated from the number of active and inactive chemicals, as shown in Table 1

Bagging is an easy-to-develop and powerful technique for class imbalance problems [28]. Figure 1a shows the effectiveness of bagging by comparing a plain neural network (NN) with a bootstrap aggregated neural network (NN-bagging) and a neural network-based representation ensemble (NN-representation ensemble). As shown in Fig. 1a, bagging improved the AUC in both ensemble techniques. As shown in Fig. 1b, the improved AUC by bagging was correlated with the imbalance ratio of the dataset (Pearson’s r=0.69, p-value= 1.1×10^−3^). The results showed greater improvement with a higher imbalance ratio.

The proposed multi-subject comprehensive ensemble combines all models regardless of learning method or representation: 12 models consisting of the unique combinations of representations (PubChem, ECFP, and MACCS) and learning methods (RF, SVM, GBM, and NN) and the newly proposed SMILES-NN model. All ensembles except for the last column combined the various models by uniform averaging. The comprehensive ensemble outperformed all limited ensemble approaches based on average combining.

In terms of the combination technique, we compared simple uniform averaging with the proposed meta-learning techniques in both comprehensive ensembles. The results of the comprehensive ensemble from Table 2 are presented in the second to the last column of Table 4. The last column in Table 4 shows the performance comparison between meta-learning and the comprehensive ensemble. The multi-task neural networks [10] achieved state-of-the-art performance on 19 PubChem bioassays with performance measurement of the AUC. As shown in Table 5, our approach outperformed multi-task learning in 13 out of 19 PubChem bioassays. From “Convolutional and recurrent neural networks” section, this result was statistically significant at a p-value of 3.9×10^−8^ in 13 out of 19 datasets and resulted in a higher mean AUC value for the meta-learning network than for the multi-task network.

**Table 5:** Performance comparison between multi-task [10] and meta-learning neural networks

Assay ID	Multi-task	Proposed (Meta-learning)
1851_1a2	0.938	**0.943**
1851_2c19	0.903	**0.908**
1851_2c9	0.907	**0.908**
1851_2d6	0.861	**0.892**
1851_3a4	0.897	**0.920**
1915	0.750	**0.764**
2358	0.751	**0.807**
463213	0.676	**0.694**
463215	0.654	0.634
488912	0.816	0.700
488915	0.873	0.739
488917	0.894	0.841
488918	0.842	0.801
492992	0.829	**0.862**
504607	0.670	**0.726**
624504	0.889	**0.904**
651739	0.825	0.809
651744	0.900	**0.909**
652065	0.792	**0.832**

The mean AUC values for both neural networks are shown (bold: top AUC on each dataset)

#### Performance comparison on other dataset

The Drug Therapeutics Program (DTP) AIDS Antiviral Screen developed an HIV dataset for over 40,000 compounds. These results are categorized into three groups: confirmed inactive (CI), confirmed active (CA) and confirmed moderately active (CM). Following previous research [29], we also combined the latter two labels (CA and CM), resulting it a classification task to discriminate inactive and active.

We evaluated our meta-learning neural network on the HIV dataset following identical experimental settings as described in MoleculeNet [29]. The HIV dataset was divided by scaffold-based splitting into training, validation, and test sets at a ratio of 80:10:10. Scaffold-based splitting separates structurally different molecules into different subgroups [29]. For the performance metrics, we used AU-ROC, accuracy, Matthews correlation coefficient (MCC), and F1-score. Accuracy, MCC, and F1-score were defined as follows:

$$\begin{array}{*{20}l} &\texttt{Accuracy} = \frac{TP+TN}{TP+TN+FP+FN} \\ &\texttt{MCC} = \frac{TP*TN-FP*FN}{\sqrt{(TP+FP)(TP+FN)(TN+FP)(TN+FN)}} \\ &\texttt{F1-score} = \frac{2TP}{2TP+FP+FN} \\ \end{array} $$

where *TP*, *FP*, *FN*, and *TN* represent the number of true positives, false positives, false negatives, and true negatives, respectively. Table 6 shows the results for the comparison between multi-task [10] and meta-learning on the various performance metrics. For meta-learning, we applied our neural networks described in Section 2.3.4 to the multi-task neural network. We repeated the experiments 100 times and calculated the mean test score. In terms of AU-ROC, both neural networks performed similarly, however, meta-learning outperformed multi-task learning in other metrics.

**Table 6:** Performance comparison with Multi-task neural networks [10] on HIV datasets [29]

	AUC	Accuracy	MCC	F1-score
Multi-task [10]	0.714 ±0.007	0.947 ±0.009	0.260 ±0.020	0.972 ±0.005
Meta-learning	0.714 ±0.007	0.964 ±0.001	0.269 ±0.026	0.982 ±0.001

The table shows the average test set of various measures for Multi-task neural networks and Meta-learning neural networks

### Meta-learning and interpretation of model importance

We made a final decision through meta-learning using the predictions from independent first-level models as input. Any learning algorithm could be used as a meta-learner. We used SVM, which achieved the highest average AUC value in further experiments compared with NN, RF, GBM, and ordinary regression.

**Fig. 2:**
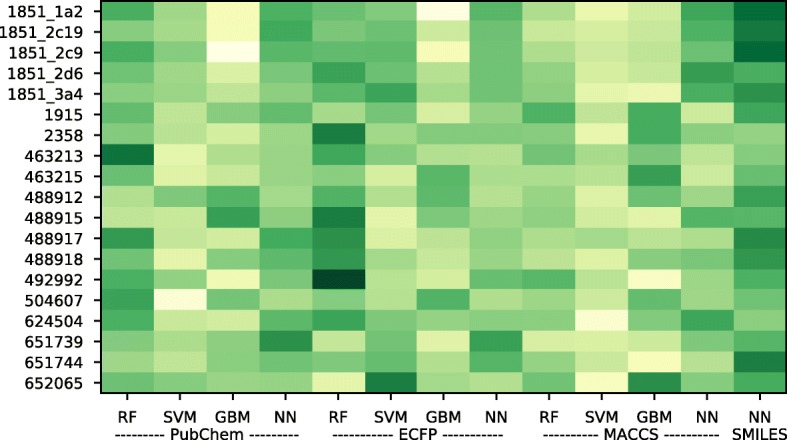
Interpretation of model importance through meta-learning. Weights through meta-learning were used to interpret model importance. Darker green indicates a highly weighted and significant model, while lighter yellow indicates a less weighted and less significant model

We interpreted the importance of the models through their learned weights. In the process of meta-learning, a weight is assigned to each model, and this weight could be interpreted as the model importance. As shown in Fig. 2, the degree of darkness for each method is slightly different depending on the dataset, just as the best prediction method and representation depends on the datasets (Table 2). A darker color indicates a higher weight and importance. PubChem-SVM, ECFP-GBM, and MACCS-SVM showed low importance, while SMILES-NN and ECFP-RF showed high importance throughout the dataset. The SMILES-NN model did not show as high a performance as an individual model, but it was regarded as the most important model.

## Discussion

Ensemble learning can improve predictability, but it requires a set of diversified hypotheses; bagging requires a set of randomly sampled datasets, a method ensemble needs to exploit diverse learning methods, and a representation ensemble needs to prepare diversified input representations. A comprehensive ensemble requires diversified datasets, methods, and representations across multi-subjects; thus, it has difficulties in preparation and learning efficiency for these hypotheses.

Diversity is a crucial condition for ensemble learning. RF was superior to NN among the individual models, but NN outperformed RF in the representation ensemble. This is presumably due to model variation diversities caused by random initialization and random dropout of the neural network. In addition to model variation diversity, SMILES seems to contribute to ensemble representation diversity. The SMILES-based model did not show impressive results as an individual model, but it was considered the most important predictor when combined.

The proposed comprehensive ensemble exploits diversities across multi-subjects and exhibits improved predictability compared to the individual models. In particular, the neural network and SMILES contribute to diversity and are considered important factors when combined. However, the proposed ensemble approach has difficulties associated with these diversities.

## Conclusions

We proposed a multi-subject comprehensive ensemble due to the difficulties and importance of QSAR problems. In our experiments, the proposed ensemble method consistently outperformed all individual models, and it exhibited superiority over limited subject ensemble approaches and uniform averaging. As part of our future work, we will focus on analyzing as few hypotheses as possible or combinations of hypotheses while maintaining the ensemble effect.

## Methods

### Ensemble learning

Ensemble learning builds a set of diversified models and combines them. Theoretically and empirically, numerous studies have demonstrated that ensemble learning usually yields higher accuracy than individual models [11, 12, 30–32]; a collection of weak models (inducers) can be combined to produce a single strong ensemble model.

#### Framework

Ensemble learning can be divided into *independent* and *dependent* frameworks for building ensembles [33]. In the independent framework, also called the randomization-based approach, individual inducers can be trained independently in parallel. On the other hand, in the dependent framework (also called the boosting-based approach), base inducers are affected sequentially by previous inducers. In terms of individual learning, we used both independent and dependent frameworks, *e.g.*, RF and gradient boosting, respectively. In terms of combining learning, we treated the individual inducers independently.

#### Diversity

Diversity is well known as a crucial condition for ensemble learning [34, 35]. Diversity leads to uncorrelated inducers, which in turn improves the final prediction performance [36]. In this paper, we focus on the following three types of diversity.

- *Dataset diversity*
  The original dataset can be diversified by sampling. Random sampling with replacement (bootstrapping) from an original dataset can generate multiple datasets with different levels of variation. If the original and bootstrap datasets are the same size (*n*), the bootstrap datasets are expected to have ($1-\frac {1}{e}$) (≈63.2*%* for *n*) unique samples in the original data, with the remainder being duplicated. Dataset variation results in different prediction, even with the same algorithm, which produces *homogeneous* base inducers. Bagging (bootstrap aggregating) belongs to this category and is known to improve unstable or relatively large variance-error factors [37].
- *Learning method diversity*
  Diverse learning algorithms that produce *heterogeneous* inducers yield different predictions for the same problem. Combining the predictions from *heterogeneous* inducers leads to improved performance that is difficult to achieve with a single inducer. Ensemble combining of diverse methods is prevalently used as a final technique in competitions, that presented in [10]. We attempted to combine popular learning methods, including random forest (RF) [8, 38], support vector machine (SVM) [39], gradient boosting machine (GBM) [40], and neural network (NN).
- *Input representation diversity*
  Drugs (chemical compounds) can be expressed with diverse representations. The diversified input representations produce different types of input features and lead to different predictions. [21] demonstrated improved performance by applying ensemble learning to a diverse set of molecular fingerprints. We used diverse representations from PubChem [22], ECFP [23], and MACCS [24] fingerprints and from a simplified molecular input line entry system (SMILES) [25].

#### Combining a set of models

For the final decision, ensemble learning should combine predictions from multiple inducers. There are two main combination methods: weighting (non-learning) and meta-learning. Weighting method, such as majority voting and averaging, have been frequently used for their convenience and are useful for homogeneous inducers. Meta-learning methods, such as *stacking* [41], are a learning-based methods (second-level learning) that use predictions from first-level inducers and are usually employed in heterogeneous inducers. For example, let *f*_*θ*_ be a classifier of an individual QSAR classifier with parameter *θ*, trained for a single subject (drug-specific task) *p*(*X*) with dataset *X* that outputs *y* given an input *x*. The optimal *θ* can be achieved by

1 $$ \theta^{*} = \text{argmax}_{\theta}\mathbb{E}_{(x,y)\in X}[p_{\theta}(y|x)]  $$

Then, the second-level learning will learn to maximize output *y* by learning how to update the individual QSAR classifier $\phantom {\dot {i}\!}f_{\theta ^{*}}$. “First-level: individual learning” section details the first-level learning and, “Second-level: combined learning” section details the second-level learning.

### Chemical compound representation

Chemical compounds can be expressed with various types of chemical descriptors that represent their structural information. One representative type of chemical compound descriptor is a molecular fingerprint. Molecular fingerprints are encoded representations of a molecular structure as a bit-string; these have been studied and used in drug discovery for a long time. Depending on the transformation to a bit-string, there are several types of molecular fingerprints: structure key-based, topological or path-based, circular, and hybrid [42]. Structure key-based fingerprints, such as PubChem [22] and MACCS [24], encode molecular structures based on the presence of substructures or features. Circular fingerprints, such as ECFP [23], encode molecular structures based on hashing fragments up to a specific radius.

Another chemical compound representation is the simplified molecular-input line-entry system (SMILES) [25], which is a string type notation expressing a chemical compound structure with characters, *e.g.*, C,O, or N for atoms, = for bonds, and (,) for a ring structure. SMILES is generated by the symbol nodes encountered in a 2D structure in a depth-first search in terms of a graph-based computational procedure. The generated SMILES can be reconverted into a 2D or 3D representation of the chemical compound.

Examples of SMILES and molecular fingerprints of leucine, which is an essential amino acid for hemoglobin formation, are as follows:

- SMILES string: CC(C)CC(C(=O)O)N
- PubChem fingerprint: 1,1,0,0,0,0,0,0,0,1,1,0,0,0,1,0,⋯
- ECFP fingerprint: 0,1,0,0,0,0,0,0,0,0,0,0,0,0,0,0,⋯
- MACCS fingerprint: 0,0,0,0,0,0,0,0,0,0,0,0,0,0,0,0,⋯
  (Most values in this molecular fingerprint are zero).

**Fig. 3:**
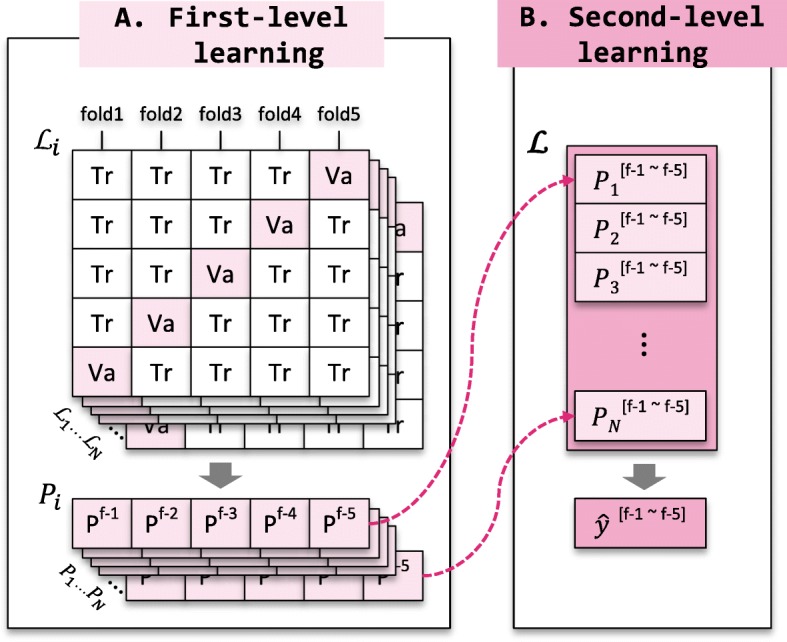
Learning procedure of the proposed comprehensive ensemble. The individual *i*-th learning algorithm $\mathcal {L}_{i}$ outputs its prediction probability *P*_*i*_ for the training dataset through 5-fold cross-validation. The *n* diverse learning algorithms produce *n* prediction probabilities (*P*_1_,*P*_2_,⋯,*P*_*n*_). The probabilities are concatenated and then used as input to the second-level learning algorithm $\boldsymbol {\mathcal {L}}$, which makes a final decision $\hat {y}$. **a** First-level learning. **b** Second-level learning

Figure 3 shows the two-levels of learning procedure. First-level learning is an individual learning level from diversified learning algorithms and chemical compound representations. The prediction probabilities produced from first-level learning models are used as inputs for second-level learning. Second-level learning makes the final decision by learning the importance of individual models produced from the first-level predictions.

### Notation

The notation used in our paper is as follows:

- **x**: preprocessed chemical compound-representation input, where **x** can be a particular type of molecular fingerprints or SMILES.
- **h**: hidden representation
- $\mathcal {L}$: first-level individual learning algorithm ($\mathcal {L}_{i}$: *i*-th algorithm, *i*={1,⋯,*n*})
- $\boldsymbol {\mathcal {L}}$: second-level learning algorithm
- *P*: predicted probability from the individual model (*P*_*i*_: predicted probability from the $\mathcal {L}_{i}$)
- $\hat {y}$: final predicted decision from the second-level learning
- *σ*: activation function (*σ*_*s*_: sigmoid, *σ*_*r*_: rectified linear unit (ReLU), and *σ*_*t*_: hyperbolic tangent)
- *n*: total number of individual algorithms

### First-level: individual learning

With a combination of learning algorithms and chemical compound input representations, we generated thirteen kinds of individual learning models: nine models from conventional machine learning methods, three models from a plain feed-forward neural network, and one model from the 1D-CNN and RNN-based newly proposed neural network model.

#### Conventional machine learning methods

Among the conventional machine learning methods, we used SVM, RF, and GBM with three types of molecular fingerprints, resulting in nine combination models consisting of all unique pairs of learning algorithms (SVM, RF, and GBM) and fingerprints (PubChem, ECFP, and MACCS). We set the penalty parameter to 0.05 for the linear SVM, and the number of estimators was set to 100 for RF and GBM based on a grid search and experimental efficiency. The prediction probabilities from these learning methods are used as inputs for second-level learning. However, SVM outputs a signed distance to the hyperplane rather than a probability. Thus, we applied a probability calibration method to convert the SVM results into probabilistic outputs.

#### Plain feed-forward neural network

We used a plain feed-forward neural network (NN) for the vector-type fingerprints: PubChem-NN, ECFP-NN, and MACCS-NN. The neural network structure consists of three fully connected layers (Fcl) with 512, 64, and 1 units in each layer and using, the ReLU, tanh, and sigmoid activation functions, respectively,

2 $$ P= \sigma_{s}(\mathbf{Fcl}(\sigma_{t}(\mathbf{Fcl}(\sigma_{r}(\mathbf{Fcl}(\mathbf{x})))))).   $$

The sigmoid activation function outputs a probability for binary classification. We used the Adam optimizer [43] with binary cross-entropy loss (learning rate: 0.001, epoch: 30, and mini-batch size: 256).

#### Convolutional and recurrent neural networks

To learn key features through end-to-end neural network learning automatically, we used a SMILES string as input and exploited the neural network structures of the 1D-CNNs and RNNs. A CNN is used to recognize the short-term dependencies, and an RNN is used as the next layer to learn long-term dependencies from the recognized local patterns.

**Fig. 4:**
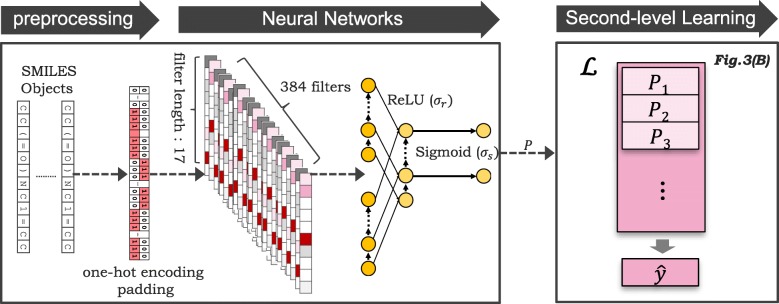
Proposed CNN + RNN model. The input SMILES strings are converted with one-hot encoding and truncated to a maximum length of 100. The preprocessed input is subsequently fed to the CNN layer without pooling, and the outputs are directly fed into the GRU layer

As illustrated in Fig. 4 of the preprocessing step, the input SMILES strings were preprocessed with one-hot encoding [44–46], which sets only the corresponding symbol to 1 and others to 0. The input is truncated/padded to a maximum length of 100. We only consider the most frequent nine characters in SMILES and treat the remaining symbols as OTHERS, thus the encoding dimension was reduced to 10.

As illustrated in Fig. 4 of the neural networks step, the preprocessed input **x** was fed into the CNN layer without pooling (CNN filter length: 17, number of filters: 384). Then, the outputs from the CNN were fed into the GRU layer (dimension: 9, structure: many-to-many).

3 $$ \mathbf{h}= \sigma_{t}(\mathbf{GRU}(\sigma_{r}(\mathbf{Conv}(\mathbf{x})))),   $$

where **h** is the output of GRU layer, *σ*_*r*_ is the ReLU, and *σ*_*t*_ is the hyperbolic tangent. The output **h** was flattened and then fed into a fully connected neural network.

4 $$ P= \sigma_{s}(\mathbf{Fcl}(\sigma_{r}(\mathbf{Fcl}(\mathbf{h}_{\text{\texttt{flatten}}})))),   $$

where *P* is the output probability from the sigmoid activation function for binary classification. The output *P* is subsequently used for second-level learning as in the last step in Fig. 4.

We used dropout for each layer (CNN: 0.9, RNN: 0.6, first Fcl: 0.6) and an Adam optimizer (learning rate: 0.001, epoch: 120, mini-batch size: 256) with binary cross-entropy. Most of these hyperparameters were empirically determined.

### Second-level: combined learning

We combined the first-level predictions generated from the set of individual models to obtain the final decision.

We have *n* individual learning algorithms $\mathcal {L}_{i}$, where *i*={1,⋯,*n*}, and the *i*-th model outputs the prediction probability *P*_*i*_ for a given **x**. We can determine the final prediction $\hat {y}$ by weighting, *w*_*i*_:

5 $$ \hat{y}=\sum_{i=1}^{n}w_{i}P_{i}(\mathbf{x}),   $$

where if the weight *w*_*i*_=1/*n*,∀*i* indicates, *uniform averaging*.

As another technique, we can combine the first-level output predictions through meta-learning. The performance of individual methods varies depending on each dataset as shown in “Performance comparison with individual models” section; there is no invincible universal method. The learned weights from the individual models are applied to the corresponding datasets. Thus, we use learning based combining methods (meta-learning) rather than simple averaging or voting.

6 $$\begin{array}{*{20}l} \hat{y}&=\boldsymbol{\mathcal{L}}(\mathcal{L}_{1}(\mathbf{x}), \mathcal{L}_{2}(\mathbf{x}),\cdots,\mathcal{L}_{n}(\mathbf{x})) \end{array} $$

7 $$\begin{array}{*{20}l} &=\boldsymbol{\mathcal{L}} \left ([P_{1}, P_{2}, \cdots, P_{n}] \right),  \end{array} $$

where $\boldsymbol {\mathcal {L}}$ is a second-level learning algorithm, and any machine learning method can be applied this level. All *P*_*i*_, where *i*={1,2,⋯,*n*} are concatenated and used as inputs. The model importance imposes a weight *w*_*i*_ on *P*_*i*_ and is determined through meta-learning.

## Data Availability

The datasets generated and/or analyzed during the current study are available at http://data.snu.ac.kr/QSAR/.

## Abbreviations

1D-CNNs: One-dimensional convolutional neural networks
AU-PRC: Area under the curve of the receiver operating characteristic curve
AUC: Area under the curve
GBM: Gradient boosting machine
GRU: Gated recurrent units
HTS: High throughput screening
MTNN: Multi-task neural networks
NN: Neural network
QSAR: Quantitative structure-activity relationship
RF: Random forest
RNNs: Recurrent neural network
SMILES: simplified molecular-input line-entry system
SVM: Support vector machine
